# Detection of functional PTEN lipid phosphatase protein and enzyme activity in squamous cell carcinomas of the head andeck, despite loss of heterozygosity at this locus

**DOI:** 10.1054/bjoc.2001.1848

**Published:** 2001-06

**Authors:** J Snaddon, E K Parkinson, J A Craft, C Bartholomew, R Fulton

**Affiliations:** School of Biological and Biomedical Sciences, Glasgow Caledonian University, Cowcaddens Rd, Glasgow, G4 0BA; Beatson Institute for Cancer Research, Wolfson Laboratory for Molecular Pathology, Glasgow

**Keywords:** PTEN, LOH, carcinoma, P13K

## Abstract

The human tumour suppressor gene PTEN located at 10q23 is mutated in a variety of tumour types particularly metastatic cases and in the germline of some individuals with Cowdens cancer predisposition syndrome. We have assessed the status of PTEN and associated pathways in cell lines derived from 19 squamous cell carcinomas of the head and neck. Loss of heterozygosity is evident at, or close to the PTEN gene in 5 cases, however there were no mutations in the remaining alleles. Furthermore by Western analysis PTEN protein levels are normal in all of these SCC-HN tumours and cell lines. To assess the possibility that PTEN may be inactivated by another mechanism, we characterized lipid phosphatase levels and from a specific PIP3 biochemical assay it is clear that PTEN is functionally active in all 19 human SCCs. Our data strongly suggest the possibility that a tumour suppressor gene associated with development of SCC-HN, other than PTEN, is located in this chromosomal region. This gene does not appear to be MXI-1, which has been implicated in some other human tumour types. PTEN is an important negative regulator of PI3Kinase, of which subunit alpha is frequently amplified in SCC-HN. To examine the possibility that PI3K is upregulated by amplification in this tumour set we assessed the phosphorylation status of Akt, a downstream target of PI3K. In all cases there is no detectable increase in Akt phosphorylation. Therefore there is no detectable defect in the PI3K pathway in SCC-HN suggesting that the reason for 3q26.3 over-representation may be due to genes other than PI3K110α. © 2001 Cancer Research Campaign http://www.bjcancer.com


*British Journal of Cancer* (2001) **84**(12), 1630–1634
© 2001 Cancer Research Campaign
doi: 10.1054/ bjoc.2001.1848, available online at http://www.idealibrary.com on IDEAL®

http://www.bjcancer.com

### Detection of functional PTEN lipid phosphatase protein and enzyme activity in squamous cell carcinomas of the head and neck, despite loss of heterozygosity at this locus

**J Snaddon[1], EK Parkinson[2], JA Craft[1], C Bartholomew[1] and R Fulton[1]**

[1]School of Biological and Biomedical Sciences, Glasgow Caledonian University, Cowcaddens Rd, Glasgow, G4 0BA; [2]Beatson Institute for Cancer Research, Wolfson Laboratory for Molecular Pathology, Glasgow

**Summary** The human tumour suppressor gene PTEN located at 10q23 is mutated in a variety of tumour types particularly metastatic cases and in the germline of some individuals with Cowdens cancer predisposition syndrome. We have assessed the status of PTEN and associated pathways in cell lines derived from 19 squamous cell carcinomas of the head and neck. Loss of heterozygosity is evident at, or close to the PTEN gene in 5 cases, however there were no mutations in the remaining alleles. Furthermore by Western analysis PTEN protein levels are normal in all of these SCC-HN tumours and cell lines. To assess the possibility that PTEN may be inactivated by another mechanism, we characterized lipid phosphatase levels and from a specific PIP3 biochemical assay it is clear that PTEN is functionally active in all 19 human SCCs. Our data strongly suggest the possibility that a tumour suppressor gene associated with development of SCC-HN, other than PTEN, is located in this chromosomal region. This gene does not appear to be MXI-1, which has been implicated in some other human tumour types. PTEN is an important negative regulator of PI3Kinase, of which subunit alpha is frequently amplified in SCC-HN. To examine the possibility that PI3K is upregulated by amplification in this tumour set we assessed the phosphorylation status of Akt, a downstream target of PI3K. In all cases there is no detectable increase in Akt phosphorylation. Therefore there is no detectable defect in the PI3K pathway in SCC-HN suggesting that the reason for 3q26.3 over-representation may be due to genes other than PI3K110α. © 2001 Cancer Research Campaign http://www.bjcancer.com

**Keywords**: PTEN; LOH; carcinoma; P13K

Squamous cell carcinoma of the head and neck (SCC-HN) has consistent deletions at a number of chromosomal positions including a high frequency at 10q (Bockmuhl et al, 2000). The tumour suppressor gene PTEN (**p**hosphatase and **ten**sin homologue located on chromosome **10**) at 10q23 is mutated in a variety of tumour types and in the germline of some individuals with Cowdens disease who are predisposed to cancer (Marsh et al, 1999). PTEN, also known as MMAC1 (mutated in multiple advanced cancers) and Tep1, has been reported to be mutated and deleted in a variety of carcinomas and glioblastomas, particularly advanced and metastatic cases (Ali et al, 1999). The gene encodes a dual specificity phosphatase a key biological function of which is to remove phosphate residues from PIP3 (Maehama and Dixon, 1999). The lipid phosphatase activity of PTEN thus decreases the abundance of a central constituent in a positive cell proliferation pathway, resulting in negative regulation of cell division (Vasquez and Sellers, 2000). Therefore loss and mutation of the gene leading to inactive PTEN protein would provide an obvious growth advantage.

Conflicting data have so far been published on the role of PTEN loss in SCC-HN. In one study of aerodigestive SCCs, 41% of cases had PTEN loss of heterozygosity (LOH) with a quarter of these having mutation of the second allele or homozygous deletion (Okami et al, 1998). Shao et al (1998) found 6 LOHs and 3 mutations in 19 SCC-HN whereas in another analysis of 21 HN SCCs no PTEN loss or mutation was found (Kubo et al, 1999). It is also possible that the p110α subunit of P13 kinase gene itself is over-expressed or amplified in SSC-HN, as its chromosomal location, 3q26.3, is often over-represented in this tumour type (Speicher et al, 1995) and the p110α subunit of P13K is over-represented and overexpressed in ovarian (Shayesteh et al, 1999) and cervical tumours (Ma et al, 2000). Thus the P13 kinase pathway can be upregulated by alternative mechanisms.

Therefore PTEN and its associated pathways were investigated to establish any involvement in our panel of SCC-HN cell lines, which are more amenable to biochemical analysis than tumour material. Loss of heterozygosity, allele mutations and the presence of a functional gene product were assessed in 19 independent carcinoma lines and fibroblast lines from the same individual, where available. Lack of MXI-1 involvement had already been established (unpublished results). Although over a third of our lines had LOH of one PTEN allele there were no mutations in the second allele. Furthermore PTEN and phospho Akt protein levels have been assessed and along with an estimation of the PTEN lipid phosphatase activity these were all shown to be normal. These results suggest that perturbation of the P13 kinase pathway is uncommon in SCC-HN.

*Received 31 October 2000*
*Revised 21 March 2001*
*Accepted 27 March 2001*

*Correspondence to:* R Fulton

### MATERIALS AND METHODS

### Cell lines

All the cell lines were cultured using Swiss 3T3 feeders in DMEM media supplemented with 10% FBS and 0.4 μg $ml^{-1}$ hydrocortisone. The SCC-HN lines are named SCC or BICR (B) and were derived as described previously in Rheinwald and Beckett (1981) and Edington et al (1995) respectively. Control lines included normal keratinocytes (HEK), SV40 transformed HEK cells and a PTEN negative melanoma cell line SK Mel 23 (Yamashita et al, 1999). Matched fibroblasts were derived from the same patients with the original tumours (F) i.e. 6B and 6F, for example, are tumour and control lines from the same individual.

### LOH analysis by microsatellite PCR

Microsatellite PCR reactions contained 1.5 mM $MgCl_2$, 0.2 mM each dNTP, 0.2 μM each primer, 40 ng template DNA, 0.5 U Taq polymerase (Promega), and 1 × Taq buffer (Promega) in a 12.5 μl volume. For these reactions, a 5 minute denaturation step at 95˚C was followed by 35 cycles of 30 seconds at 95˚C, 75 seconds at 52˚C and 30 seconds at 72˚C. This was followed by a final extension step of 5 minutes at 72˚C.

| Locus | Primer | Sequence |
|---|---|---|
| D10S185 | 6a: | 5′-TCCTATGCTTTCATTTGCCA-3′ |
| | 6m: | 5′-CAAGACACACGATGTGCCAG-3′ |
| D10S2491 | 265AF: | 5′-GTTAGATAGAGTACCTGCACTC-3′ |
| (PTEN) | 265AR: | 5′-TTATAAGGACTGAGTGAGGGA-3′ |
| D10S192 | 094tcaa: | 5′TTATACTAGGAAACAAGGCTTACC-3′ |
| | 094tcab: | 5′-GGGCTTAAATGAATGACAC-3′ |
| MXI-1 PS1 | MXI-1.R: | 5′-TTAAATACAGGTCCTCTGACCC- 3′ |
| | MXI1.F: | 5′-GGTTACTCCCGTGCCAGTGT-3′ |
| D10S587 | 6816: | 5′-CCCAGATTCATGGCTTTC-3′ |
| | 6817: | 5′-TTCTGCTGACACACGGGC-3′ |
| D10S222 | 3017: | 5′-TGGAAACCTACCGAATGGA-3′ |
| | 3018: | 5′-TCTAACTGTGGATTGAAGCGAC-3′ |

### Denaturing polycrylamide gel electrophoresis

For resolution of microsatellite markers, 6% denaturing acrylamide gels (6% acrylamide 1 × TBE, 7 M urea) were used. Following the addition of 1/2 volume loading dye (95% formamide, 20 mM EDTA, 0.25% xylene cyanol, 0.25% bromophenol blue), the samples were heat denatured at 94˚C for 5 min prior to loading. The Protean II system (Biorad) was used, and the gels electrophoresed at 300 V for at least 4 hours.

Visualization was by silver staining.

### PCR and sequencing of PTEN Exons

Each exon was amplified individually from genomic DNA using intron specific primer sequences (Reisinger et al, 1997). PCR reactions contained 1.5 mM $MgCl_2$, 0.2 mM each dNTP, 1 μM each primer, 50 ng template DNA, 1 U Taq polymerase (Promega), and 1 × Taq buffer (Promega) in a 25 μ1 volume. Reaction conditions were as follows: 95˚C for 5 min, then 7 cycles of 30 s at 95˚C, 30 s at 55˚C, decreased by 1˚ per cycle, and 1 min at 72˚C. This was followed by 30 cycles of 1 min each at 95˚C, 48˚C and 72˚C respectively and a final 6 min extension at 72˚C.

Each product was then electrophoresed on 1% agarose, visualized by ethidium bromide staining, and the bands excised and purified using the Geneclean II kit (Bio 101).

50 ng of each template was initially sequenced using the Sequenase PCR product Sequencing kit (Amersham). Sequence was confirmed by cloning each product into pGEM-T Easy vector (Promega) and sequencing in both directions using M13 Universal and Reverse primers with the Sequenase V2.0 sequencing kit (Amersham).

### Western blotting

$1 \times 10^6$ cells were lysed in 100μl RIPA buffer (1 × PBS, 1% Nonidet P-40, 0.5% sodium deoxycholate, 0.1% SDS) containing 5 μg $ml^{-1}$ aprotonin, 5 μg $ml^{-1}$ leupeptin, 5 μg $ml^{-1}$ pepstatin A, 1 mM benzamidine and 50 μg $ml^{-1}$ PMSF. Lysates were left on ice for 20 min, then centrifuged at 13 000 rpm for 15 min. Cleared supernatants were transferred to fresh microfuge tubes, and the protein concentration of each determined by the Bradford Assay.

30 μg each whole cell extract was mixed with an equal volume of sample buffer (250 mM Tris, 20 mM DTT, 2% SDS, 0.01% Bromophenol blue, 10% glycerol) and denatured at 95˚C for 5 min, prior to separation by discontinuous SDS-PAGE on a 10% gel. Following electrophoresis, proteins were transferred onto nitrocellulose, and blocked in 1 × TBS (10 mM Tris, pH 8, 150 mM NaCl) containing 5% skimmed-milk powder for at least one hour. Primary antibody was added at the appropriate dilution in TBST (1 × TBS, 0.1% Tween 20) containing 0.5% milk, and incubated for an hour with shaking. Blots were washed 4 × 5 min in 1 × TBST prior to addition of the relevant HRP-conjugated secondary antibody (see below for antibody details) diluted in 1 × TBST, 0.5% milk. Incubation for one hour at room temperature was followed by 4 washes in TBST with a final wash in TBS. Detection of bound antibody was achieved using Luminol Reagent (Santa-Cruz) and exposure to Hyperfilm ECL (Amersham).

Blots were stripped in 0.2 M Glycine, pH 2.6, 0.1% SDS for one hour at room temperature, washed several times in TBST then re-blocked in TBS/5% milk for an hour before re-probing with a different primary antibody.

| Antibody | Dilution | Secondary antibody |
|---|---|---|
| PTEN A2B1 (Santa-Cruz) 1/5000 | 1/750 | Goat anti-mouse (Sigma) |
| ERK2 (Transduction Labs) 1/5000 | 1/2000 | Goat anti-mouse (Sigma) |
| PhosphoSer473 AKT (New 1/5000 England Biolabs) | 1/1000 | Goat anti-rabbit (Sigma) |
| AKT (New England Biolabs) 1/5000 | 1/1000 | Goat anti-rabbit (Sigma) |

### PIP3 lipid phosphatase assays

Whole cell extracts were prepared as described above. 1 μg PTEN A2B1 antibody (Santa-Cruz) was added to each extract and incubated on a shaking platform for 1 hour at 4˚C. 20 μl Protein G PLUS-Agarose (Santa- Cruz) was added and left for a further hour with shaking. Immunoprecipitates were pelleted by centrifugation at 4000 rpm and washed twice in RIPA buffer and twice in 10 mM

© 2001 Cancer Research Campaign 

Tris pH 8, 50 mM sodium chloride, each time by resuspension in wash buffer then centrifugation as above. Immune complexes were resuspended in enzyme reaction buffer without substrate (50 mM Tris pH 8, 50 mM sodium chloride, 10 mM DTT, 10 mM magnesium chloride). This immunoprecipitated PTEN was added to prewarmed reaction buffer (50 mM Tris pH 8, 50 mM sodium chloride, 10 mM DTT, 10 mM magnesium chloride, 10 μM L-α-phosphatidylinositol-3, 4, 5-triphosphate (Calbiochem)), in a 50 μl volume. Reactions were incubated for 30 min at 37˚C, then terminated by the addition of 100 μl BIOMOL Green reagent (BIOMOL). The amount of phosphate present was determined by reading the absorbance of the samples at 630 nm following incubation at room temperature for 20 min to allow colour development. Phosphate concentrations were estimated, in triplicate experiments, by comparison to phosphate standards diluted in enzyme reaction buffer.

### RESULTS

### Detection of loss of heterozygosity within the PTEN locus

To determine whether any chromosomal loss had occurred at the PTEN locus in our panel of 19 SCC-HNs, microsatellite analysis was carried out using 6 primer pairs within the 10q23–26 locus. The analysis was performed by PCR on DNA from the tumour cell lines and direct comparisons made with matched fibroblast controls derived from the same patient, where these were available. The results of this analysis clearly revealed LOH in BICR cases 3, 6, 31, 78 and 82 at or adjacent to the PTEN marker D10S491 and these are indicated by asterisks with 3 markers illustrated in panels A, B and C in Figure 1. Two cases, BICR 63 and 68, had clear retention of heterozygosity as illustrated in Figure 1 and a further 6 cases were non-informative possibly due to homozygosity at this marker. Microsatellites from the PTEN marker and 5 other markers at this chromosomal locus were used to determine LOH status in this region as presented in Table 1. The boundaries of potential loss are outlined in bold and in BICR3, 6, 31 and 82 this loss includes the PTEN marker (Table 1).

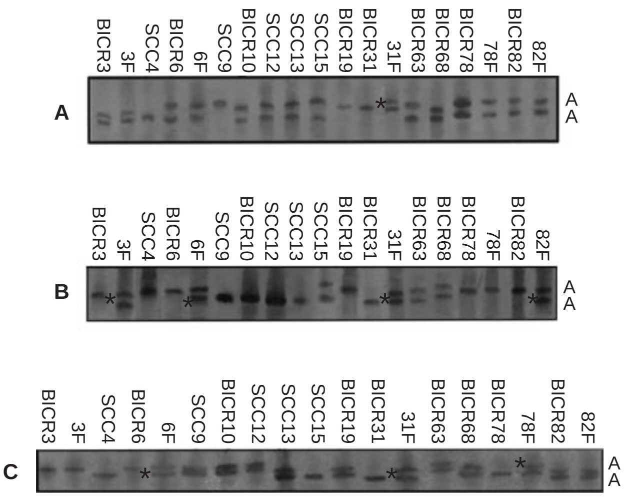

**Figure 1** Microsatellite analysis of 14 tumour cases (B and SCC) and matched controls where available (F) illustrating LOH in 5 of these. Three markers illustrated are D10S 185 (**A**), the PTEN marker D10S2491 (**B**) and marker D10S192 (**C**). Allele loss is indicated by an asterisk beside the remaining allele

**Table 1** LOH data for HN SCC tumour set

| Tumour name | Locus D10S185 | D10S491 (PTEN) | D10S491 | D10S491 | PS1 (MXI-1 | D10S491 |
|---|---|---|---|---|---|---|
| BICR 3 | ○ | ● | ⊘ | ● | ⊘ | ● |
| BICR 6 | ○ | ● | ● | ○ | ○ | ○ |
| BICR 19 | ⊘ | ⊘ | ○ | ○ | ○ | ⊞ |
| BICR 31 | ● | ● | ● | ⊘ | ● | ● |
| BICR 63 | ○ | ○ | ○ | ○ | ○ | ○ |
| BICR 68 | ○ | ○ | ○ | ○ | ○ | ○ |
| BICR 78 | ○ | ⊘ | ● | ○ | ⊘ | ○ |
| BICR 82 | ○ | ● | ○ | ○ | ○ | ○ |

KEY:
Loss of heterozygosity ●; Retention of heyerozygosity ○;
Non informative ⊘; Tumour names are listed down the left hand side and microsatellite markers along the top with potential regions of loss boxed in bold.

### Screening for mutation in the PTEN second allele where evidence suggests loss of the first

Loss of DNA in the vicinity of the PTEN gene suggests the presence of a tumour suppressor gene in this region, if PTEN was the candidate then it would be accompanied by inactivation of the second allele. With PTEN there are technical difficulties in searching for second allele mutations due to the presence of a highly conserved pseudogene which directly interferes with the PCR process when exon specific primers are used, therefore intron specific primers have been designed (Risinger et al, 1997). These primers allowed specific exon amplification from the functional gene. Sequencing of the products from all 9 exons revealed a complete lack of any somatic mutations in our LOH tumour DNAs from cases BICR 3, 6, 31, 78 and 82.

### Assessment of PTEN and phosphoAkt protein levels by Western analysis

Methylation, rather than mutation, can be a mechanism of PTEN inactivation (Whang et al, 1998) and may have explained the lack of second allele mutations. To rule out the possibility of another gene inactivation mechanism initially PTEN protein levels were determined by Western analysis. The recent availability of a C-terminal antibody to PTEN allowed clear characterization of protein levels in this SCC-HN series. All 19 lines were analysed by Western blotting, 17 of these are illustrated in Figure 2 with PTEN in the upper panel. Inclusion of SKMe123 (Right hand lane, Figure 2), the well characterized PTEN negative, melanoma cell line, as a control allowed us to contrast the abundant PTEN levels in our tumour set. These significant levels of PTEN were also found in keratinocyte control line HEK and SV40 transformed keratinocytes and correlated inversely with the abundance of phospho Akt (Figure 2, panel below PTEN). Again in contrast to this in the SKMe123 line phospho Akt levels were high (right hand lane same panel). The presence of phospho Akt is a clear indicator of an active P13 Kinase pathway, which is only found in the absence of PTEN. As shown total Akt levels are uniform throughout and densitometry verifies this visual analysis. Bradfords assays were used to load an accurate 30 ug per lane. As a loading control we used the ERK2 (Cobb and Goldsmith, 1995), antibody and this clearly illustrates the presence of protein equivalents in every lane in a reproducible manner (bottom panel Figure 2).

 *© 2001 Cancer Research Campaign*

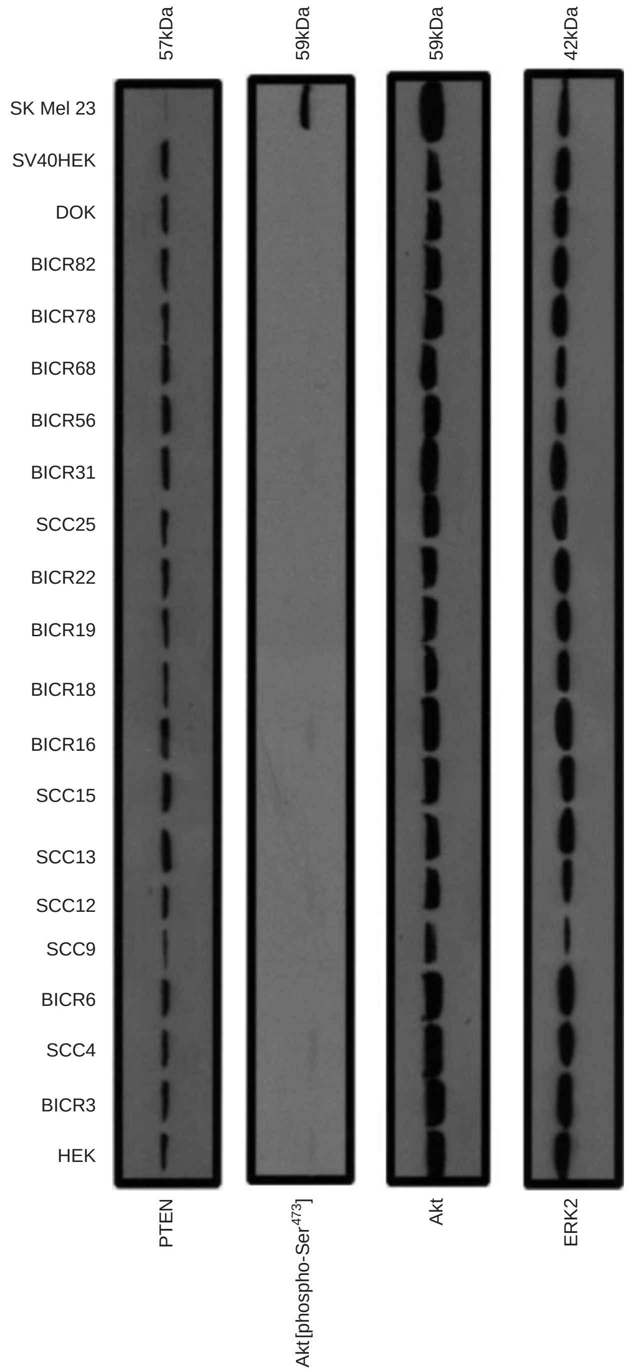

**Figure 2** Western analysis of a sample of 17 of our tumour lines and 4 controls with cell lines labelled along the top. The antibodies are indicated beside the appropriate panel down the left hand side with the protein size on the RHS

### PIP3 lipid phosphatase activity in this tumour series

Having confirmed that PTEN protein was present in our head and neck squamous cell carcinoma lines we wished to determine whether this protein was indeed functionally active. To do this we carried out a colorimetric, PIP3-specific, lipid phosphatase assay. Included in this assay was the PTEN-negative SKMe123 line and normal human keratinocytes as negative and positive controls respectively. The assays were optimized using time course experiments and carried out in triplicate. Immunoprecipitated PTEN protein was assayed and found to be active in all SCC and BICR lines examined with a range of phosphatase activity reproducibly represented by 280–385 pmoles of phosphate released (Figure 3 and Table 2). Basal levels of PTEN activity were detected in the SKMe123 line, the mock immunoprecipitation and the control with buffer and substrate alone (top 3 bars in Figure 3).

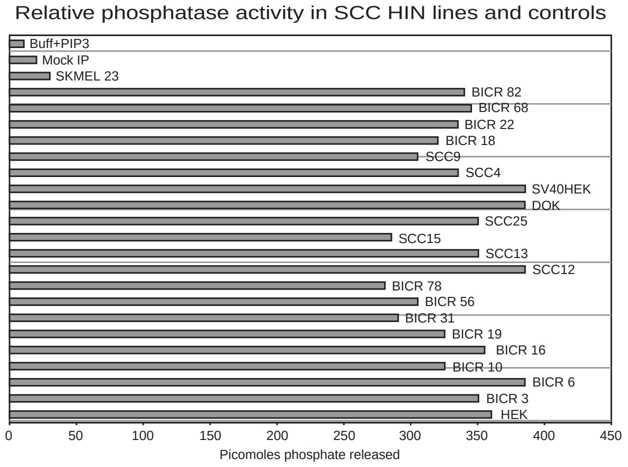

**Figure 3** Graphical representation of phosphatase assay data. Each bar is labelled with the representative assay contents and the Y axis indicates picomoles of phosphate released in the PIP3 colorimetric assay with immunoprecipitated PTEN protein

### DISCUSSION

Chromosomal loss in cancers is very common with consistent patterns giving strong clues to tumour suppressor gene location.

**Table 2** Statistical analysis of phosphate release in PTEN assay

| Cell line | Mean released phosphate (pmol) | SEM (+/–) | *P* |
|---|---|---|---|
| HEK | 373 | 14.814 | 1.000 |
| BICR3 | 357 | 8.819 | 0.400 |
| SCC4 | 322 | 4.410 | 0.063 |
| BICR6 | 345 | 13.229 | 0.228 |
| SCC9 | 330 | 7.638 | 0.081 |
| BICR10 | 352 | 6.667 | 0.281 |
| SCC12 | 362 | 22.048 | 0.686 |
| SCC13 | 355 | 10.408 | 0.375 |
| SCC15 | 320 | 2.887 | 0.064 |
| BICR16 | 358 | 9.280 | 0.448 |
| BICR18 | 332 | 11.667 | 0.095 |
| BICR19 | 350 | 8.660 | 0.261 |
| BICR22 | 340 | 5.000 | 0.143 |
| SCC25 | 367 | 11.667 | 0.742 |
| BICR31 | 327 | 4.410 | 0.077 |
| BICR56 | 333 | 6.667 | 0.097 |
| BICR68 | 350 | 10.000 | 0.271 |
| BICR78 | 333 | 6.667 | 0.097 |
| BICR82 | 348 | 11.667 | 0.259 |
| DOK | 382 | 21.279 | 0.766 |
| SV40HEK | 397 | 8.819 | 0.262 |
| SK Mel 23 | 27 | 3.333 | 0.001 |

Shown is the mean of the phosphate released from PIP3 by immunoprecipitated PTEN from each cell line in 3 independent experiments and the standard error of the mean (SEM) in each case. *P* is a measure of the probability that the difference in phosphate released between each cell line and HEK cells occurred by chance, calculated using the *t*-test method. Statistical significance is indicated by $P < 0.05$.

*© 2001 Cancer Research Campaign*

Loss of 10q in SCC-HN has a controversial history which led us to approach the problem using both DNA marker loss complemented with biochemical analysis of the PTEN gene product and members of the associated P13 Kinase signal transduction pathway. Loss of the chromosomal region including PTEN occurs in 26% of this tumour series which provides a strong indication that there is a tumour suppressor gene at this site. In this subset of lines all 9 exons in the remaining PTEN allele were sequenced and shown to be the same as normal. The lack of mutation in the coding exons of the second allele of PTEN suggests that this gene may not be the deletion target in this locus. However other mechanisms of gene inactivation are possible (Whang et al, 1998) and we eliminated these by detection of significant PTEN protein levels and specific lipid phosphatase activity. LOH is also seen within the MXI-1 gene in line BICR 31 however no mutation was discovered in the second allele (unpublished results) suggesting a lack of direct involvement of this gene.

Since relatively similar PTEN lipid phosphatase activities were found in all SCC tumour and normal cell lines and no significantly different levels were found in corresponding situations in which LOH was recorded, PTEN gene dosage does not appear to play a role in the immortalization of these cells. This highlights the importance of the specific functional assay, where several years ago mutations may have been artefactual or confused with the pseudogene (Rhei et al, 1997) our approach answers directly whether PTEN is inactivated or decreased in activity.

Protein phosphatases can antagonise or modulate the signals mediated by protein kinases. PTEN negatively controls the PI3 kinase signalling pathway and results in regulation of cell growth and survival. The inverse correlation between PTEN and Phospho Akt levels is well recognized in normal cells (Vasquez and Sellers, 2000), since this is a member of the signal transduction pathway switched off by functional PTEN. A lack of phospho Akt allowed us to confirm the presence of functional PTEN in our tumour lines. Also in the control, SK Mel 23, PTEN is inactive and phospho Akt levels are correspondingly high. Interestingly the low phospho Akt levels in our SCC-HN lines also suggests low PI3 Kinase levels despite the observation that the p110α subunit maps to the SCC-HN amplicon at 3q26.3. However, the lack of significantly increased Akt phosporylation suggests that PI3 kinase is not amplified in these tumours.

PTEN may affect tumour invasion and metastasis (Gasparotto et al, 1999) and also replicative senescence (Tresini et al, 1999). All the keratinocyte cultures studied are immortal but our results showing mainly low levels of Akt phosporylation, do not support a general role for PI3 kinase upregulation in the immortalization of this type. A pattern that is now emerging, and is supported by our data, suggests that PTEN loss is infrequent in SCC-HN (Chen et al, 2000) and is more commonly associated with metastatic SCCs at low frequency and as our tumour series contains mainly primary or recurrent and only one metastatic tumour (BICR 22) this could explain the lack of PTEN involvement.

In conclusion it appears likely from our data that another tumour suppressor gene located in close proximity to MXI-1 and PTEN is lost in a high proportion of head and neck squamous cell carcinomas. Alternatively, the loss of 10q23 may be co-selected with other chromosome losses in SCC-HN progression. The future characterisation of other genes near to PTEN should resolve this issue.

### ACKNOWLEDGEMENTS

Dr V Brunton Beatson Institute Glasgow – pAkt and akt antibodies. Dr M Edwards Dermatology Department Glasgow University – SK Mel 23 line.

### REFERENCES

Ali IU, Schriml LM and Dean M (1999) Mutational spectra of PTEN/MMAC1 gene: A tumor suppressor with lipid phosphatase activity. *J Nat Cancer Inst* **91**(22): 1922–1932

Bockmuhl U, Schmidt S, Peterson S and Peterson I (2000) Deletion of chromosome 10q–a marker for metastasis of head and neck carcinomas? *Laringorhinootologie* **79**(2): 81–85

Boyd J (1997) Mutaton analysis of the putative tumour supressor gene PTEN/MMAC1 in primary breast carcinomas. *Cancer Res* **57**: 3657–3659

Chen Q, Samaranayake LP, Zhou H and Xiao L (1999) Homozygous deletion of the PTEN tumor suppressor gene is not a feature in oral squamous cell carcinoma. *Oral Oncology* **36**: 95–99

Cobb MH and Goldsmith EJ (1995) *Journal of Biological Chemistry* **270**: 14843–14846

Edington K, Loughran OP, Berry IJ and Parkinson EK (1995) Cellular immortality: A late event in the progression of human squamous cell carcinoma of the head and neck associated with p53 alteration and a high frequency of allele loss. *Mol Carcinog* **13**: 254–265

Kubo Y, Urano Y, Hida Y and Arase S (1999) Lack of somatic mutation in the PTEN gene in squamous cell carcinoma of human skin. *Journal of Dermatological Science* **19**: 199–201

Ma Y-Y, Wei S-J, Lin Y-C, Lung J-C, Chang T-C, Whengpeng J, Liu JM, Yang D-M, Yang WK and Shen C-Y (2000) P13CA as an oncogene in cervical cancer. *Oncogene* **19**: 2739–2744

Maehama T and Dixon JE (1999) The tumour suppressor, PTEN/MMAC1, dephosphorylates the lipid second messenger, phosphatidylinositol 3,4, 5-trisphosphate. *The Journal of Biological Chemistry* **273**: 13375–13378

Marsh DJ, Kum JB and Lunetta KL (1999) PTEN mutation spectrum and genotype-phenotype correlations in Bannayan-Riley-Ruvalcaba syndrome suggest a single entity with Cowden syndrome. *Human molecular genetics* **8**: 1461–1472

Okami K, Wu L, Riggins G, Cairns P, Goggins M, Evron E, Halachmi N, Ahrendt SA, Reed AL, Hilgers W, Kern SE, Koch WM, Sidransky D and Jen J (1998) Analysis of PTEN/MMAC1 alterations in aerodigestive tract tumours. *Cancer Research* **58**: 509–511

Quinn AG, Sikkink S and Rees JL (1994) Basal cell carcinomas and squamous cell carcinomas of human skin show a distinct pattern of chromosome loss. *Canc Res* **54**: 4756–4759

Rhei E, Kang L, Bogomolniy F, Federici MG, Borgen PI and Boyd J (1997) Mutation analysis of the putative tumor suppressor gene PTEN/MMAC1 in primary breast carcinomas. *Cancer Research* **57**: 3657–3659

Rheinwald JG and Beckett MA (1981) Tumorigenic keratinocyte lines requiring anchorage and fibroblast support cultured from human squamous-cell carcinomas. *Cancer research* **41**: 1657–1663

Risinger JI, Hayes AK, Berchuck A and Barrett JC (1997) PTEN/MMAC1 mutations in endometrial cancers. *Cancer Research* **57**: 4736–4738

Shao X, Tandon R, Samara G, Kanki H, Yano H, Close LG, Parsons R and Sato T (1998) Mutational analysis of the PTEN gene in head and neck squamous cell carcinoma. *Int J Canc* **77**: 684–688

Shayesteh et al (1999) *Nat Genet* **21**: 99–102

Speicher MR, Howe C, Crotty P, Du Manoir S, Costa J and Ward DC (1995) Comparative genomic hybridisation detects novel deletions and amplifications in head and neck squamous cell carcinomas. *Cancer Res* **55**: 1010–1013

Tresini M, Mawal-Dewan M, Cristofolo VJ and Sell C (1998) *Cancer Res* **58**: 1–4

Vasquez F and Sellers WR (2000) The PTEN tumour suppressor protein: an antagonist of phosphoinositide signalling. *BBA* **1470**: M21–M35

Whang YE, Wu XY, Suzuki H, Reiter RE, Tran C, Vessella RL, Said JW, Isaacs WB and Sawyers CL (1998) Inactivation of the tumor suppressor PTEN/MMAC1 in advanced human prostate cancer through loss of expression. Proceedings of the National Academy of Sciences of the United states of America, 95, no.9, 5246–5250

Yamashita T, Tonoki H, Moriuchi T, Jin HY and Jimbow K (1999) Wild-type p53 induces apoptosis in pigmented melanoma cell lines SK-mel-23 and 70W without altering p21 (Wafl), Bcl-2 and Bax expression. *Journal of Investigative Dermatology* **112**(4): 701

*© 2001 Cancer Research Campaign*
